# Controlled Solvent-Free Formation of Embedded PDMS-Derived Carbon Nanodomains with Tunable Fluorescence Using Selective Laser Ablation with A Low-Power CD Laser

**DOI:** 10.3390/mi8100307

**Published:** 2017-10-14

**Authors:** María José González-Vázquez, Mathieu Hautefeuille

**Affiliations:** 1 Laboratorio Nacional de Soluciones Biomiméticas para Diagnósticoy Terapia, Facultad de Ciencias, Universidad Nacional Autónoma de México, Avenida Universidad 3000, Circuito Interior S/N, Delegación Coyoacán, Ciudad Universitaria, Mexico City C.P. 04510, Mexico; majito@ciencias.unam.mx; 2 Departamento de Física Facultad de Ciencias, Universidad Nacional Autónoma de México, Avenida Universidad 3000, Circuito Exterior S/N, Delegación Coyoacán, Ciudad Universitaria, Mexico City C.P. 04510, Mexico

**Keywords:** carbon nanodomains, fluorescence, laser ablation, poly-dimethylsiloxane (PDMS)

## Abstract

We present a study of the application of a single-step and solvent-free laser-based strategy to control the formation of polymer-derived fluorescent carbon nanodomains embedded in poly-dimethylsiloxane (PDMS) microchannels. A low-power, laser-induced microplasma was used to produce a localised combustion of a PDMS surface and confine nanocarbon byproducts within the exposed microregions. Patterns with on-demand geometries were achieved under dry environmental conditions thanks to a low-cost 3-axis CD-DVD platform motorised in a selective laser ablation fashion. The high temperature required for combustion of PDMS was achieved locally by strongly focusing the laser spot on the desired areas, and the need for high-power laser was bypassed by coating the surface with an absorbing carbon additive layer, hence making the etching of a transparent material possible. The simple and repeatable fabrication process and the spectroscopic characterisation of resulting fluorescent microregions are reported. In situ Raman and fluorescence spectroscopy were used to identify the nature of the nanoclusters left inside the modified areas and their fluorescence spectra as a function of excitation wavelength. Interestingly, the carbon nanodomains left inside the etched micropatterns showed a strong dependency on the additive materials and laser energy that were used to achieve the incandescence and etch microchannels on the surface of the polymer. This dependence on the lasing conditions indicates that our cost-effective laser ablation technique may be used to tune the nature of the polymer-derived nanocarbons, useful for photonics applications in transparent silicones in a rapid-prototyping fashion.

## 1. Introduction

In the pursuit of simpler and direct microfabrication processes that enable surface etching or tridimensional bulk patterning in a variety of polymeric materials, high-energy short-pulsed lasers have been used to produce micro and nano patterns by single step ablation [1,2,3,4]. When additive processes are sought, micro/nanoparticle laser sintering has also demonstrated promising results in miniaturisation for nanotechnology rapid-prototyping [5,6]. Recently, high-power lasers have been employed for one-step, direct micromachining of a low-cost polymer matrix of poly-dimethylsiloxane (PDMS). This technique exploits its low cost, biocompatibility and ease of use to fabricate complex tridimensional structures with a broad range of applications in microtechnology, microfluidics and photonics, showing the high potential of polymer laser processing in microtechnology [7,8,9]. Moreover, high power lasers can also be used to induce physical or chemical transformations of polymers in a controlled, localised fashion, broadening further the field of potential applications, including biological cell culture in microscaffolds, enabled by superficial laser modification [10]. In some studies, embedded nanoparticles have been induced or modified in other polymer matrices using lasers, as the local manipulation and structuration of metallic nanodomains has become important for the development of microdevices that would benefit from their optoelectronic properties [11,12]. In the last decade, various allotropes of carbon have become materials under extensive study due to the attractive optical, mechanical and physicochemical properties obtained with a variety of processes for many different applications. In particular, it has been shown that high-power lasers may be exploited to tune and stabilise the photoluminescence (PL) spectra of carbon nanodomains by adjusting their shape, dimensions, or passivation states thanks to appropriate lasing conditions or solvent selection [13,14]. However, the use of high-energy, short-pulsed lasers and platforms is usually expensive, impractical for benchtop microfabrication solutions, and it sometimes limits the control over the production of carbon nanodomains that is required in some applications. Moreover, some solvents employed to control the nature of nanocarbon fluorescence properties can be limiting in biotechnology and tissue engineering applications, especially if remaining traces enter into contact with cells or tissues. Finally, this method of obtaining the desired carbon nanodomains remains limited as it systematically requires further delicate steps such as recollection and deposition without contaminating the particles.

We have reported that it is possible to reach etching performance similar to that of high-power lasers using an affordable low-power (inferior to 100 mW), near-infrared compact-disc (CD) laser diode (780 nm). When that laser is precisely focused at the polymer sample, the addition of a thin layer of strongly-absorbing additive enables the formation of a local microplasma in polymers that are otherwise transparent at such wavelength [15]. In addition to laser microetching with a precise control of features dimensions in poly-dimethylsiloxane (PDMS) or poly-methylmethacrylate (PMMA) [16], it has also been shown that this low-cost process can be exploited for the formation of localised fluorescent nanocarbon combustion residues inside design-specific micropatterns after ablation in PDMS [17]. Also, it has been found that different types of residues were left under different laser and additive-coating conditions, pointing towards a possible control of the nature of the PDMS-derived nanocarbon after microplasma [17]. Because of this, the process of superficial laser-induced incandescence proved to be useful for controlling the production of localised fluorescent nanodomains in a PDMS matrix with a direct, in situ, and rapid-prototyping method without the need of organic solvents to control the passivation as reported elsewhere [13,14].

In this work, we present a study of the possible control of the nature of the nanocarbon residues left in PDMS after direct laser microetching by local microplasma using our commercial CD platform. As the microplasma is generated by a strong absorption of the additive carbon material that is coating the transparent polymer, we have analysed the dependency of the resulting residues found inside the micropatterns etched in PDMS with the nature of additives and laser power densities, as these parameters control the temperature reached during microplasma and may then determine the final PL properties. Indeed, it has been shown that the laser-induced incandescence threshold is strongly dependent on the dimensions and nature of the nanocarbon upon which the light is shone [18]. This localised microplasma phenomenon has been imputed to a surface plasmon in nanotubes [19]. In this particular case, very high temperatures of approximately 1750 K have been reported at the surface of the absorbing nanoparticles when 100 mW lasers were focused [20,21]. Thanks to their great reproducibility, single and multi-wall carbon nanotubes (SWCNT and MWCNT) as well as fullerenes (C60) additives were used in completely dry conditions, thus presenting an additional benefit as no liquid solvent is necessary in our case: when the laser is focused onto the additive-coated PDMS layers, it causes a localised temperature rise that can be used for precise and local etching, as absorbing the NIR wavelength generates localised laser-induced incandescence [17]. The embedded nanodomains were characterised using micro-Raman and fluorescence spectroscopy and showed a strong dependency with easily controllable conditions.

## 2. Materials and Methods

### 2.1. Samples Preparation

The production of PDMS-derived carbon nanodomains is controlled here by the formation of a localised laser-induced microplasma thanks to the strong focusing of a near infrared light (NIR) laser beam originating from an optical pickup unit (OPU) of a commercial CD-DVD burner system. This OPU was mounted on a three-axis micro-displacement platform with a motorised computer numerical control (CNC) as detailed in [15,16,17]. The system also enables the precise control of laser conditions such as pulse time, optical power density in either continuous or pulsed mode, as well as the dwell time. As previously reported, this control is important here as it has a great impact on the formation of nanostructures in local laser ablation of PDMS [16,17].

**Figure 1:**
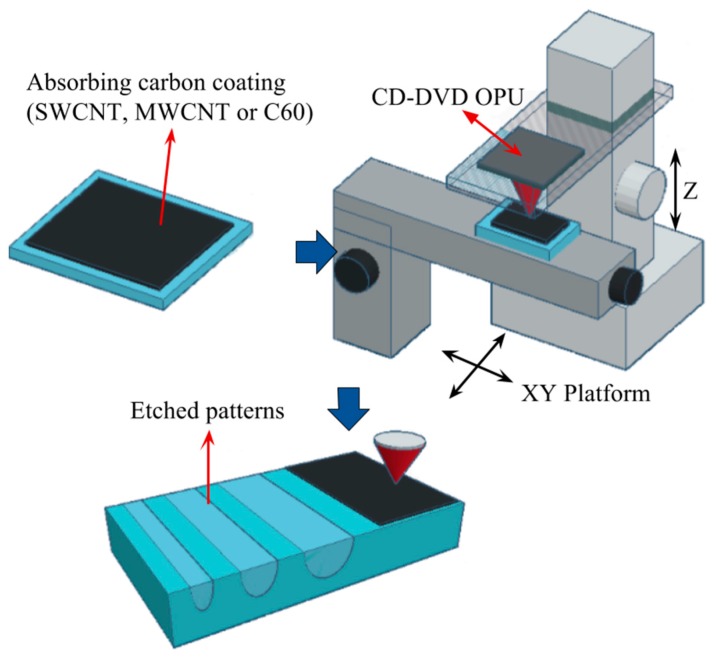
Diagram of the low-cost fabrication process used to generate laser-induced incandescence on poly-dimethylsiloxane (PDMS) coated with a nanocarbon additive.

As PDMS presents very low absorption of NIR, microplasma-based ablation was achieved by coating cured PDMS layers with absorbing carbon nanomaterials, following the process previously reported [15,17] and summarised in Figure 1. PDMS samples were prepared by mixing Sylgard^®^ 184 (Dow Corning, Midland, MI, USA) silicone elastomer base with its corresponding curing agent at a 20:1 ratio. Previous tests had indeed shown that a carbon nanopowder coating presented a better adherence and homogeneity on a less rigid PDMS surface (a 20:1 ratio represents a lower Young’s modulus than a more common 10:1 ratio). The desired mixture was then poured on clean glass microscope slides and cured for 2 h at 60 °C in a convection oven. After curing, the PDMS slabs were coated with a thin layer of carbon nanostructures that strongly absorbed laser light.

Three different types of materials were tested here in order to evaluate the influence of the coating layer that promotes the laser-induced microplasma on the resulting formation of polymer-derived nanocarbon residues inside PDMS etched features. The absorbing coating powders employed in this work were SWCNT, MWCNT and C60 used as purchased from Sigma Aldrich (St. Louis, MO, USA), and without further treatment (part numbers 773735, 698849 and 572500 respectively). A few milligrams of powder was homogeneously spread and pressed onto the clean and flat PDMS surface using a spatula and the surface was finally brushed away to guarantee a thin, homogeneous coating layer as the powders adhered to PDMS. Thermal ageing of PDMS layers on a hotplate at 150 °C for 2 h, immediately before coating, also helped lower surface tension and improve coating without modifying the process described here.

### 2.2. Laser Etching of Micropatterns

When focusing the infrared beam onto the additive layer coating the surface, there is a power density threshold (It), defined as the lower limit of laser intensity from which the micron-scale laser-induced incandescence is capable of generating the localised combustion of the polymer. Once determined experimentally, the transfer of the desired residues’ micropatterns was controlled with the programming of our platform. Although a pulsed laser regime can be used with our platform, a continuous wave (CW) mode with a 5 ms dwell time is only reported here, as the impact of pulse duration on the residue outcome was not very clear. It is thought that longer durations of the existence of the microplasma and the resulting elevated heat generated at the surface of the PDMS promoted the combustion of the silicon-based elastomer material in a very repeatable fashion. Visible residues were found in the PDMS laser-etched channels [16]. It was interesting to note that the colour of the bright visible emission of the laser-induced incandescence during the process depended strongly on the type of carbon nanomaterial coating the sample. This is probably related to the temperature of the plasma that is responsible for the ablation of the polymeric material underneath and the production of nanocarbon residues of this ablation. Previous characterisation of similar experiments had already indicated that selective laser ablation was producing nanocarbon residues inside the micron-scale laser-etched PDMS patterns using carbon nanopowder as an additive [14]. It was also shown that laser intensity may modify the nature of these residues, although there was little control on this outcome. Etching of the PDMS surface was however achieved in a very reproducible fashion following this procedure and the structures were cleaned with distilled water and isopropyl alcohol in order to remove remaining additive materials from the surrounding unexposed areas and leave the micropatterns only with the embedded nanodomains that are under study here.

All seemed to indicate that local temperature conditions might influence the formation of nanodomains and further tests were thus performed in this work with PDMS samples coated with SWCNT, MWCNT and C60. It was indeed thought that different materials would lead to a variety of absorption and clustering behaviours as different patterns, depths and widths were found in similar lasing conditions but with different materials [22]. Characterisation of micro-patterned features dimensions, such as widths and depths, were achieved with a KLA Tencor D600 Profilometer (KLA Tencor, Milpitas, CA, USA).

### 2.3. Characterisation of Nanocarbon Residues and Tunable Fluorescence

In this work, in spite of not measuring the temperature reached at the surface, we observed a strong relationship between additive materials used to ablate PDMS and the nature and fluorescence signal of polymer-derived nanodomains that are left in the channels. It has indeed been observed [17] that these residues were not washed away, as they remain strongly attached underneath the PDMS surface after ablation. Moreover, although the laser process is similar to one reported in [23], where silver nanoparticles were embedded in PDMS and remained in the same allotropic form, in our case the nanoresidues left inside the channels after the process were found to be different from the initial additive materials used to coat the surface of the elastomer [17].

Micro-Raman spectroscopy was achieved using the 532 nm laser excitation source of a Thermo Scientific DXR system (Waltham, MA, USA) to identify the carbon signature of residues in each case. Laser power at the sample was kept below 4 mW to avoid further photo-induced damage of our samples and long-exposure spectra were taken in order to confirm that the signal was not modified with time in the regions of interest. Different spectra were systematically obtained for each sample at different regions: pristine PDMS, carbon coated PDMS before laser ablation, and various areas of etched PDMS patterns obtained with different laser power densities.

Finally, fluorescence spectroscopy was carried out to verify the tunability of PL emission of the residues studied with Raman spectroscopy. To address the dependence of the emission wavelength on the nature of the residues, two different lasers were used as excitation sources: 325 nm and 405 nm. The experimental set ups are described elsewhere [24]. It is important to mention that the contribution of PDMS autofluorescence was always discarded as it was 2 to 5 times lower than that of etched PDMS.

## 3. Results

### 3.1. Etching Process Results

**Figure 2:**
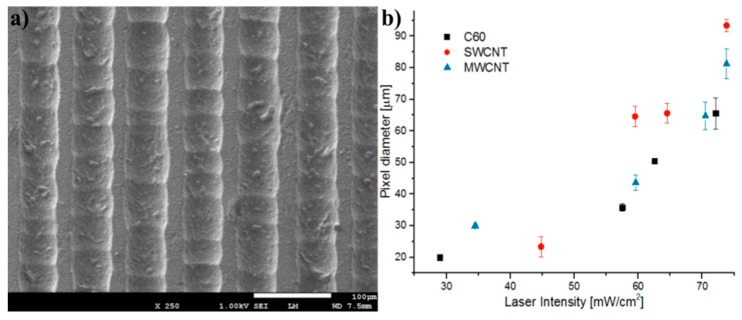
(**a**) SEM micrograph of a typical PDMS laser-etched region and (**b**) the experimental relationship between pixel resolution and laser intensity during ablation. The data presented are averaged over more than 20 values measured for five different laser intensities and the standard deviation is shown.

Figure 2 shows a scanning electron microscope (SEM) micrograph of typical laser-etched PDMS micropatterns achieved in this work (Figure 2a). It is similar to what is demonstrated in this material with high power, short-pulsed lasers. Figure 2b shows the influence of both the laser intensity and the nature of the coating additive used to establish the laser-induced incandescence on the lateral resolution of a single ablated pixel (pixel diameter) achieved after ablation. Due to the centrosymmetric geometry of circular laser ablation pixels, it is relevant to consider the diameter as the lateral characteristic feature. It is interesting to note that the relationship between the resolution and laser intensity is similar for all nanocarbon additives: the best curve-fitting shows an exponential dependency justified by the increasing intensity of the microplasma, hence local temperature, that follows the same law. Moreover, from the results it can be inferred that the type of carbon additive plays an important role in the microplasma generation, as the intensity threshold and pixel size at greatest intensities is somehow affected by the nature of the coating. This may be explained by the temperature reached for each material as their optical absorption at the laser wavelength [25] and thermal stability [26] may be responsible for the severity of the resulting combustion of PDMS, which is known to take place above 400 degrees Celsius [27].

### 3.2. Spectroscopy Results

PDMS laser ablation is caused by a localised temperature increase caused by the microplasma, which is in turn dependent on the nature of the carbon additive used to generate incandescence. In previous works, combustion of PDMS was shown to leave very stable PDMS-derived ceramic residues with photoluminescence properties at the surface of PDMS. Moreover, the nature of such residues has been found to be strongly dependent on the heat intensity and heating rates of the polymer [27,28]. At greater temperatures, commonly attained locally with laser ablation and to reach combustion, carbon nanodomains were found in the PDMS etched patterns as probable combustion residues embedded under the surface and the nature of those domains seem to depend on the laser intensity [17].

Thanks to these antecedents, and due to a possible difference in local temperatures and heating rates for each of the materials under test in this work, clearly evidenced in the variations of pixel resolution shown in the previous section, we studied the impact of the absorbent coating additives on the combustion residues. In general, our low-power laser process used to prepare photoluminescent nanocarbon in PDMS showed results very similar to a high-energy laser method where passivation permits tunability of the PL properties of the particles [13]. The PL intensity of all presented results neither decreased in time, even hours after the first irradiation, thus showing an excellent photostability, identical to that achieved in organic solvents and with lasers energies several orders of magnitude greater than ours.

**Figure 3:**
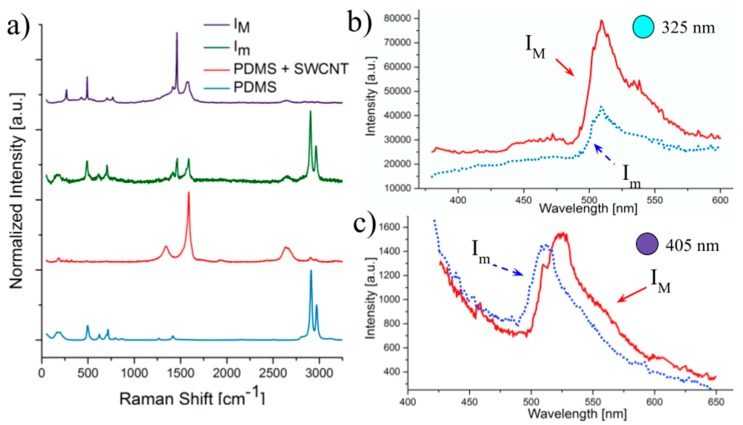
Comparison of Raman spectra (**a**) and Fluorescence emission spectra (**b**,**c**) of modified PDMS zones previously coated with single wall carbon nanotubes (SWCNT) at different wavelengths (**b**) for 325 nm excitation source and (**c**) for 405 nm excitation source.

**Figure 4:**
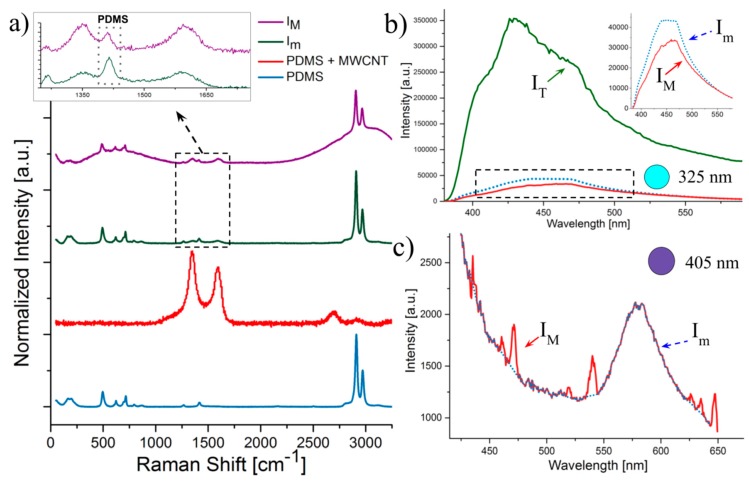
(**a**) Comparison of Raman spectra and Fluorescence emission spectra (**b**,**c**) of modified PDMS zones previously coated with multi-wall carbon nanotubes (MWCNT) at different wavelengths (**b**) for 325 nm excitation source and (**c**) for 405 nm excitation source.

**Figure 5:**
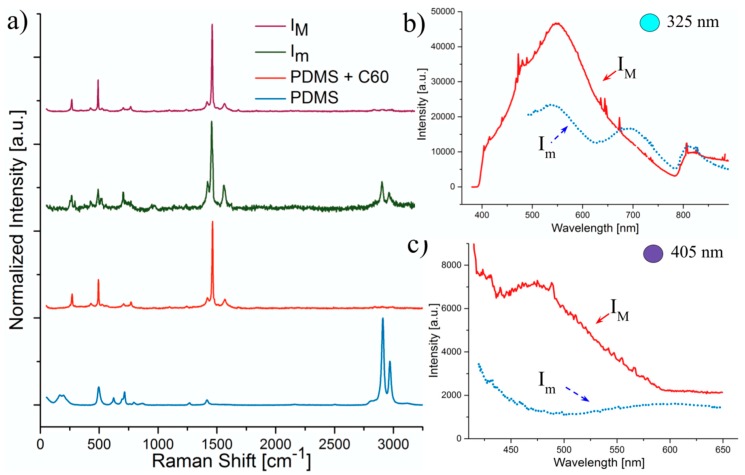
(**a**) Comparison of Raman spectra and Fluorescence emission spectra (**b**,**c**) of modified PDMS zones previously coated with fullerenes (C60) at different wavelengths (**b**) for 325 nm excitation source and (**c**) for 405 nm excitation source.

Figure 3, Figure 4 and Figure 5 show the PL and Raman spectra obtained for pristine PDMS and regions modified by laser exposure for SWCNT, MWCNT and C60 respectively. PL results are presented at two different excitation wavelengths, to show the dependence of emission on excitation energy. Moreover, two different characteristic laser intensities I_m_ (60 mW/cm^2^) and I_M_ (75 mW/cm^2^) were studied in order to analyse the influence of the laser energy on the nature of carbon nanodomains and their resulting PL. Table 1, Table 2 and Table 3 present the position of D and G bands obtained from the Raman spectra as well as the average I_D_/I_G_ ratios for the different laser power densities.

In the interpretation of the Raman spectra of laser-modified PDMS, our main interest lies in the region ranging from 1300–1700 cm^−1^, where the characteristic signal of carbonaceous traces can be found [29,30], and PDMS characteristic peaks are situated around 490 cm^−1^ and 700 cm^−1^ where the C–Si–C symmetric stretching mode appears [31,32]. It is important to notice that the characteristic band of CH_3_ asymmetric bending from PDMS around ~1411–1418 cm^−1^ is perfectly distinguishable from that of carbon, known as the D band, and although this band was found on most of the spectra in the range of 1455-1460 cm^−1^, on some of them it could be found around 1338-1343 cm^−1^. In our work, and as we mentioned in a previous report [17], no trace of SiC or SiCO were found, although it is reported that high-power laser pulses produce such ceramics [33]. This could be explained by differences between the final temperature and heat rates in both experiments. In general, for all materials used here as additives, it is clear from the Raman spectra that the ablation residues are carbon nanodomains, evidenced by the presence and intensity of the D and G bands, and that PDMS, in which they are embedded, is clearly modified by the process.

#### 3.2.1. SWCNT

In the particular case of SWCNT, there is a recurrent shift of the D band position visible after irradiation from 1350 cm^−1^ to 1460 cm^−1^. It is certainly caused by a change in diameter of the nanotubes left in PDMS after laser ablation and even more probably a dispersion of nanotubes with several dimensions as the D band is divided into two peaks. The existence of the G band, characteristic of these nanoparticles, remains visible although it decreases when laser energy increases, indicating that residues as still SWCNTs. Table 1 shows also a clear growth of the I_D_/I_G_ ratio with an increasing laser intensity, demonstrating the gradual formation of larger, more ordered carbon crystals, similar to what was reported elsewhere for higher energies [33]. Also, it can be seen that PDMS is strongly modified by our method, as can be seen in the characteristic PDMS bands. The one at 496 cm^−1^ corresponding to Si–O–Si symmetric stretching seems to be reduced after laser ablation, while Si–CH_3_ symmetric rocking at 687 cm^−1^ and Si–C symmetric stretching at 715 cm^−1^ double peak is deformed into one single peak shifted to ~707 cm^−1^. More remarkably, the intensity of the two bands at ~2905 cm^−1^ and ~2970 cm^−1^, corresponding to symmetric and asymmetric stretching of methyl group, clearly decreases relative to the carbon bands when laser power density increases, showing that the residues are almost exclusively nanocarbon.

Figure 3b,c, shows the fluorescence spectra of samples etched with SWCNT and excited at 325 nm and 405 nm respectively. The PL emission is clearly dependent on the excitation wavelength, suggesting a passivation of the nanodomains evidenced with the Raman spectra [34]. For each wavelength, there is also a dependence on the irradiation energy as the full-width at half maximum (FWHM) and maximum emission wavelength (λ_m_) at I_m_ and I_M_ are different. A narrower FWHM of ~60 nm was indeed obtained at greater energy irradiation, while it was ~84 nm under lower energy, suggesting that residues are less pure or more dispersed when PDMS was irradiated at lower energies. In the case of the 405 nm excitation a red shift of λ_M_ was observed, from ~512 nm to ~528 nm, for I_m_ and I_M_ respectively. This red edge excitation (REE) shift may be explained by a deeper embedment of the nanodomains inside PDMS after irradiation under greater energies [10] and it is consistent with the observations of the Raman spectra.

#### 3.2.2. MWCNT

Figure 4 presents Raman and PL emission spectra obtained for PDMS coated with MWCNT. In this case, all irradiated regions presented an intense fluorescence signal mounted on top of the regions corresponding to pristine PDMS, while acquiring Raman spectra. Also, the Raman signal of carbon regions was considerably less intense and quite difficult to visualise and analyse in comparison with the samples irradiated with the other two additives. A zoom on the region specific to carbonaceous domains showed the existence of D and G bands with no apparent shift of their respective position, nor intensity ratios, as shown in Table 2. Finally, it is clear that the PDMS signal remained in all spectra, possibly indicating that the polymer is less affected structurally when this additive is employed.

Regarding the fluorescence spectra of the PDMS etched regions using MWCNT as an additive, there is also a strong dependence of the PL emission wavelength with the excitation source, suggesting again a passivation of the residues inside PDMS after ablation. In this case, the fluorescence signal obtained after etching at I_T_ is also presented, as it is interesting to remark that fluorescence intensity decreased when laser etching power increased. It also appears that the spectrum obtained at I_T_ corresponds to a combination of pure MWCNT (ca. 430 nm) with another signal, visible as a shoulder around 460 nm, corresponding to what was registered at I_M_ and I_m_. Also, the signal of PDMS etched at I_T_ presented an intensity 8 times greater than what was typically obtained with other materials or with greater laser-etching power densities. This phenomenon, only observed with MWCNT, may be explained by a greater depth of embedment indicated by a slight REE shift and also seems to reflect a material modification such as oxidation or passivation caused by laser etching [35]. However, under 405 nm excitation, multiple discrete, narrow emission peaks are visible, mounted on the spectrum at greater laser intensity. This phenomenon is probably related to the formation of multiple-size or multiple-nature nanodomains inside PDMS and is consistent with the previous observation of a strong fluorescence in the Raman spectrum for I_M_.

#### 3.2.3. C60

Finally, Figure 5a shows the Raman spectra of PDMS etched with C60 as the coating additive. It is interesting to note that in this case the energy of the irradiation did not seem to affect greatly the nature of the byproducts of the ablation. As in the previous spectra, the characteristic peaks of PDMS are affected by laser ablation but only slightly. When looking closely at the relative intensity of what can be described as the D and G bands (these exact names are not in the literature) versus those characteristic of PDMS it even seems that the material is not modified and that the C60 particles remain intact inside the material without affecting them greatly. Indeed, the bands found at ∼1465 cm^−1^ and ∼1563 cm^−1^ and characteristic of C60 are left unchanged in their positions. This could be explained by the great thermal stability of C60 [36,37], that could then be dispersed locally inside the polymer after etching. However, the relative intensities that are under study here show that there is a modification of the material under laser irradiation. At greater intensities of the irradiation laser, the resulting Raman spectra are even similar to that of the additive used to coat PDMS while nanodomains with a broader D band are left at lower energies. Table 3 shows a great modification of the I_D_/I_G_ ratio between the two different laser intensities: while this ratio was of approximately 10 for the pristine material, it was reduced to nearly one third at lower intensity and then increased to 6 at greater energies. Moreover, the broader D band at lower ablation intensities divided in two close peaks. This phenomenon is most probably caused by a different microplasma temperature or cooling time [38] that slightly modified the residues without changing its intrinsic nature. This assumption has been confirmed by the PL properties of the nanoresidues obtained under these different conditions, as described in the following section.

Figure 5b,c show the fluorescence spectra from the laser modified PDMS regions formerly covered with C60. Again, the dependency of emission spectrum with the excitation wavelength is apparent but the spectra clearly differ less than for carbon nanotubes. In this case, as the Raman spectra seem to indicate that the particles and PDMS are not modified, the level of passivation is probably much lower for this material. Besides, it is very interesting to remark in Figure 5b that the regions irradiated with greater power present a broader emission spectrum and only one single peak around 547 nm, while lower-power-modified PDMS shows two additional peaks at 690 nm and 810 nm. This behaviour is similar to a different study where the temperature at which the C60 particles are formed determines their emission spectrum and the three peaks disappear at greater temperatures [36,37]. This seems to confirm our previous observation of Raman spectra indicating that C60 particles do not remain exactly intact after the ablation process but that they are heated up to a point at which their PL property is modified. The blue shift at greater laser power under a 405 nm excitation is also consistent with this hypothesis.

## 4. Conclusions

In this paper, we have presented a simple, alternative method for producing photoluminescent carbon nanodomains embedded and passivated in PDMS in a controllable, localised fashion, using a very low-cost and low-power CD-DVD pickup head. Thanks to a laser-induced incandescent microplasma obtained by focusing the laser diode beam onto a thin, strongly absorbent nanocarbon coating, ablation on this transparent material was possible. Also, polymer-derived carbon nanodomains were left as combustion residues inside the resulting etched regions. We showed that these byproducts were different from the coating materials used to produce localised ablation and that it was possible to obtain excitation-wavelength-dependent photoluminescence by controlling the nature of the coating and the laser energy. The results presented here are very similar to what was reported with high-power short-pulsed lasers. They offer the advantage of being a solvent-free passivation without weakening photostability, as the PL observed here lasted for several hours without losing intensity thanks to the embedment of the nanodomains inside the transparent PDMS. Raman and fluorescence spectra demonstrated a certain control of PL properties of the PDMS combustion byproducts thanks to a proper choice of coating materials and laser power density. All this indicates that the local temperature attained during ablation is a key parameter in obtaining carbon nanoresidues in PDMS, which will need further study. The potential impact of environmental conditions during fabrication—such as the presence of oxidative gases—should also be studied as oxygen and nitrogen are known to affect PDMS combustion and the nature of its residues.

## Figures and Tables

**Table 1:** Average position for D and G bands for each laser power density used for modifying the sample coated with SWCNT and their respective I_D_/I_G_.

Laser Power Density	D Band Position (cm^−1^)	G Band Position (cm^−1^)	I_D_/I_G_
I_m_	1460 (±2.5)	1588 (±1.7)	0.9 (±0.4)
I_M_	1458 (±2.8)	1588 (±2.3)	2.3 (±0.9)

**Table 2:** Average position for D and G bands for each laser power density used for modifying the sample coated with multi-wall carbon nanotubes (MWCNT) and their respective I_D_/I_G_.

Laser Power Density	D Band Position (cm^−1^)	G Band Position (cm^−1^)	I_D_/I_G_
I_m_	1351 (±1.4)	1593 (±1.8)	0.8 (±0.1)
I_M_	1350 (±4.6)	1595 (±0.2)	0.9 (±0.1)

**Table 3:** Average position for D and G bands for each laser power density used for modifying the sample coated with MWCNT and their respective I_D_/I_G_.

Laser Power Density	D Band Position (cm^−1^)	G Band Position (cm^−1^)	I_D_/I_G_
I_m_	1463 (±1.3)	1569 (±1.7)	3.8 (±0.8)
I_M_	1461 (±1.4)	1564 (±2.5)	6 (±0.6)
